# Climate change worry among nurses and their hope levels for climate change prevention

**DOI:** 10.1186/s12912-024-02067-9

**Published:** 2024-06-21

**Authors:** Songül Duran, Serap Kaynak

**Affiliations:** 1 Izmir Demokrasi University, Health Services Vocational College, Izmir, Türkiye; 2 Balıkesir University Faculty of Health, Department of Child Health Nursing, Balıkesir, Türkiye

**Keywords:** Climate change worry, Climate crisis, Climate change hope, Nursing

## Abstract

**Aim:**

This research aimed to determine nurses’ climate change worry, their level of hope for climate change prevention, and the relationship between climate change worry and hope for climate change.

**Background:**

Nurses are healthcare professionals actively involved in the fight against climate change. However, their close involvement with the issue can also increase their own climate change worry. Therefore, it is important to maintain high levels of hope among nurses in preventing climate change.

**Methods:**

This descriptive cross-sectional study was conducted with nurses working at a university hospital.

**Results:**

The average score on the Climate Change Worry Scale for nurses was 29.22 ± 9.33, with sub-dimensions scores as follows: personal-sphere will and way 10.96 ± 2.09; collective sphere will 18.36 ± 3.39; lack of will and way dimensions 10.40 ± 2.48. The average score on the climate change hope scale was 39.73 ± 5.52. A statistically significant positive relationship was found between age and the climate change worry scale (*r* = 0.169, *p* = 0.020) as well as climate change hope (*r* = 0.148, *p* = 0.041).

**Conclusion:**

The research findings indicate that nurses have a high level of climate change worry, but they also have a high level of hope in preventing climate change. It is considered essential to address the concerns of nurses who are actively combating the climate crisis.

## Background

The health consequences of climate change manifest as increasing ambient temperatures, severe weather events, sea level rise and emissions affecting air quality [1]. Extreme heat, air pollution, increased allergens, severe weather, impacts on water and food quality and supply, disasters and environmental degradation, including wildfires, hurricanes, and water leaks, are climate changes that affect human health [2]. One of the most concerning effects of climate change is its potential destructive impact on human health [3]. According to the World Health Organization (WHO), a warmer and more variable climate can lead to contamination of food by certain air pollutants, an increased likelihood of extreme heat, and endanger agricultural production in some developing countries [4]. The Lancet reports emphasize that as a consequence of global warming, there will be significant health impacts, including increased rates of malnutrition due to reduced crop yields and rising food prices, higher rates of infectious diseases, elevated respiratory illness rates due to air pollution, increased traumatic injuries due to more frequent extreme weather events, and the subsequent challenges [5]. Climate change significantly affects a wide range of health conditions, including asthma, domestic violence, gastrointestinal diseases, vector-borne illnesses, premature birth, and heart-related deaths [6]. Mental health professionals warn of a hidden psychological cost that will gradually increase over time as a result of the impacts of extreme and erratic weather conditions on society. This cost is expected to manifest in rising rates of depression, anxiety, post-traumatic stress disorder, substance abuse, domestic violence, divorce, homicide, and suicide [7].

Many people experience high levels of worry about climate change [8]. Climate change worry reflects an individual’s active and emotional involvement with the topic of climate change, as well as their personal distress over its consequences. This, in turn, serves as a motivating factor for the individual to take action on climate change [9]. Climate change worry contributes to environmentally friendly, climate-friendly behaviors or intentions, and actions that support climate initiatives [10]. However, excessive worrying can hinder efforts to adapt to climate change and lead to negative emotions such as tension and distress, resulting in decreased ability to solve problems [11]. As a result of not addressing large-scale climate change issues, climate change worries can become chronic and adaptation can become more difficult. This can lead to anxiety and/or depression [12]. Especially among youth and young adults, climate worry in the absence of hope leads to negative mental and emotional consequences [13].

Ona of the important elements in the fight against climate change is hope [14]. Hope is a source of motivation to activate a behavior [15]. It has been emphasized that people with high hope are more likely to be actively involved in mitigating and adapting to climate change [16]. Faced with the threat of climate change, healthcare professionals can better serve themselves, patients, and society by deciding to nurture hope [17].

The responsibility of nurses in promoting human health is closely intertwined with their role in advocating for a sustainable climate [18]. Nurses must grasp the interplay between individuals and their environment; an individual’s well-being is intricately connected to their surroundings. Comprehensive progress in human health and overall well-being necessitates a consideration of environmental health [19]. Health professionals, when dealing with climate change or when this issue comes up in any way, can do the following: emphasize the relationship between health and climate change, advocate for environmentally sustainable healthcare services, support the health benefits of policies aimed at reducing potential harm, provide healthcare services to affected communities, and contribute to preventing climate change [20]. Nurses are in a position to prepare for and respond to the health challenges that may be caused by increasing extreme weather events [21]. Nurses can also educate individuals and society in many areas as health educators [22]. Many nursing interventions can be applied, such as preparing the elderly and young people in disadvantaged groups for disaster events, improving mental health and well-being by motivating people to utilize outdoor spaces [23]. It has been observed that healthcare professionals in the field of nursing can actively contribute to preventive mental healthcare, health education, raising awareness, and advocating for policies addressing health concerns associated with climate change [2].

The International Council of Nurses (ICN) has stated that nurses have a dual responsibility to both safeguard and improve their patients’ health and address climate-related concerns, including climate mitigation (efforts to reduce or prevent greenhouse gas emissions) and climate adaptation (reducing sensitivity to adverse effects) [24]. They are actively engaged in addressing the health repercussions of climate change [24]. It has been noted that the adverse effects of climate change on health are more severe for vulnerable groups, and nursing interventions can be undertaken to address these risks [25]. While there have been studies regarding nurses’ perceptions of the effects of climate change [24, 26], no studies have been found that specifically address nurses’ own climate change worry and their levels of hope for climate change prevention. People experience fear, anxiety, or despair for issues that have not yet occurred in the future [27]. The increased awareness of nurses who recognize the issue of climate change, make efforts towards it, and intervene when necessary, can also raise their own concerns. It is believed that after addressing and improving their own health and climate change worry, nurses can also benefit others in this regard.

This research aimed to determine nurses’ climate change worry, their levels of hope for climate change prevention, and the relationship between climate change worry and the levels of hope for climate change.

## Methods

### Design, setting and sampling

This research follows a descriptive cross-sectional design. The study was conducted with nurses working at a university hospital from June to August 2023. The research population consisted of 262 nurses working in the hospital. When the sample calculation was made with a margin of error of 0.05 and a confidence interval of 95%, the number of samples was determined as 162. Taking into account the potential for data loss due to incomplete surveys or other similar issues, 190 nurses were reached.

Inclusion criteria: The criteria for participation in the research involved voluntarily completing the surveys and having a minimum of three months of experience working as a nurse.

Exclusion criteria: Those who failed to complete the questionnaires.

#### Data collection

The researchers visited the hospital and obtained written permission from the hospital management. After explaining the study’s objectives to the participants and obtaining their informed consent, the researcher instructed them to complete the survey forms. Data collection involved gathering demographic information, using the climate change worry scale, and the hope to prevent climate change scale. The survey was distributed to nurses in clinics in sealed envelopes in the morning, and preliminary information about the study was provided. The survey forms were collected back from those who had completed them before the end of the workday. Participants completed the surveys in a private room without the presence of any research team members. The survey forms do not contain any names. The informed consent form, which does include names, is collected separately from the survey. Nurses who filled out the research forms delivered the surveys to the researcher by hand.

#### Questionnaires

##### Demographic characteristics

Questions about topics such as age, gender, marital status etc. which are thought to be closely related to the level of global climate change worry and hope, are included in this section.

##### Climate change worry scale

The scale was originally developed by Stewart (2021) [11]. The Turkish validity and reliability study of the scale were conducted by Özbay and Alci [27]. The scale consists of 10 items with a five-point Likert-type rating. The scale has a single-factor structure, and its Cronbach’s alpha value is 0.9 [27].

##### Hope to prevent climate change scale

The adaptation of the scale, originally developed by Li and Monroe (2017) [16], to Turkish was conducted by Gezer and İlhan (2020) [28]. This scale is of the five-point Likert type and consists of 11 items with three subscales: individual hope, social hope, and hopelessness. Scores on this scale range from a minimum of 11 to a maximum of 55 points. A high score on the scale indicates that an individual has a high level of hope to prevent climate change. In the reliability analysis, it was determined that Cronbach alpha values were between 0.56 and 0.74 and composite reliability coefficients were between 0.58 and 0.87. Since all of these correlation values were above the 0.30 criterion, it was reported that the items in the scale were distinctive [28].

#### Statistical analysis

The data were analyzed using the SPSS (Statistical Package for the Social Sciences) software package (version 16.0, SPSS Inc., Chicago, IL, USA). Categorical variables were presented using frequencies (percentages). The relationship between scales was tested using Spearman correlation analysis. The significance level was set at 0.05.

## Results

### Participant characteristics

In the study, 76.8% of the participants are female, and 51.6% are married. 76.8% of them have a university degree or higher education level. 58.4% of the nurses have children, and 83.7% have a moderate economic status. 54.7% of the participants stated that they do not collect waste at home. The average age of the nurses is 30.87 ± 6.45, and the average years of work experience is 9.09 ± 6.06.4.2 Participants’ Average Scores on the Climate Change Worry Scale and Climate Change Hope Scale (Table 1).

**Table 1:** Sociodemographic characteristics of participatans (*n* = 190)

Characteristics	*n*	%
Gender		
Female	146	76.8
Male	44	23.2
**Marital status**		
Married	98	51.6
Single	92	48.4
**Education level**		
Secondary school	44	23.2
High school or higher level	146	76.8
**Having a child**		
Yes	79	41.6
No	111	58.4
**Economic status**		
Low level	31	16.3
Moderate level	159	83.7
**Collect waste at home**		
Yes	86	45.3
No	104	54.7
	**Mean**	**Standart Deviation**
**Age**	30.87	6.45
**Work experience (year)**	9.09	6.06

The mean score of the nurses on the climate change worry scale is 29.22 ± 9.33, with sub-dimensions of personal-sphere willpower and waypower at 10.96 ± 2.09, collective sphere willpower and waypower at 18.36 ± 3.39, and lack of willpower and waypower dimensions at 10.40 ± 2.48 points. The average score on the climate change hope scale is 39.73 ± 5.52 (Table 2).

**Table 2:** Mean scores of participants in climate change worry scale and climate change hope scale (*n* = 190)

Scales	Mean ± SD
**Climate Change Worry Scale**	29.22 ± 9.33
Personal-sphere willpower and waypower	10.96 ± 2.09
Collective-sphere willpower and waypower	18.36 ± 3.39
Lack of willpower and waypower dimensions	10.40 ± 2.48
**Climate Change Hope Scale**	39.73 ± 5.52

*Abbreviation* SD, standard deviation

### Correlations between climate change worry scale, climate change hope, age, and service years

It was determined that there was a statistically significant positive relationship between age and Climate Change Worry Scale (*r* = 0.169, *p* = 0.020) and Climate Change Hope (*r* = 0.148, *p* = 0.041). The study did not find a significant relationship between the length of time spent working and the levels of worry or hope regarding climate change among the nurses who participated in the study. It was determined that there was a statistically significant relationship (*r* = 0.457, *p* = 0.000) between the Climate Change Hope Scale and the Climate Worry Scale (Table 3).

**Table 3:** Correlations with the climate change worry scale and climate change hope, age and working years

		Climate change worry scale	Climate change hope
Age	**r**	0.169*	0.148*
	**p**	**0.020**	**0.041**
Working years	**r**	0.103	0.041
	**p**	0.157	0.574
Climate change hope	**r**	0.457**	1
	**P**	**0.000**	

* *p* < 0.05, ** *p* < 0.000

## Discussion

This research aimed to determine the levels of climate change worry and hope for climate change prevention among nurses, as well as the socio-demographic factors influencing these. It is appropriate to have a certain level of concern regarding climate change, reflecting a realistic threat perception. It is known that a certain level of concern can functionally prepare individuals to cope effectively [29]. In the study, nurses’ climate concern scale score was found to be high at 29.22 ± 9.33. Tuckett and colleagues determined in their research that nurses identified global warming (“climate change,” “greenhouse gases,” “ozone,” “CO_2_ emissions”) as the most important environmental issue [30]. In a study that determined the level of concern regarding climate change, it was found that nursing, social service, and physical therapy students received higher scores [31]. In a study, it was found that healthcare professionals perceive climate change as a public health threat [32]. A similar score of 31.3 ± 8.5 was found in a study conducted in Türkiye among individuals aged 18–65 on this topic [10]. Mat et al. determined this score as 33.72 ± 7.83 in nursing students [15]. In a study conducted in Türkiye, it was determined that the women who participated in the research were concerned about climate change [33]. Fertelli et al. determined the climate anxiety scale score in nurses as 35.36 ± 10.51 [14]. These results are similar to the scores we determined. It is thought that increasing global warming may have increased the anxiety level of nurses about climate change. Nurses’ high anxiety level can motivate and mobilize them. However, if this anxiety negatively affects the functionality of nurses, it may be beneficial to take the necessary precautions and provide psychosocial support to these people. Nurses at the clinical level play a crucial role in identifying and addressing the mental health effects of climate change, as well as being the first responders to increased cases resulting from extreme weather events [34]. In the literature, the role of nurses in the global climate crisis was mostly covered, and nurses’ own anxiety and hope levels regarding climate change were not determined. However, the primary condition for nurses to take an active role in this issue is that they have the potential to control their own anxiety. Therefore, it is believed that it is essential to assess and address nurses’ own levels of concern. Additionally, nurses’ hope levels for climate change prevention can facilitate more positive behaviors while actively participating in the climate crisis.

Individuals with a sense of hope generally experience improved well-being compared to those lacking hope. Given that hope often motivates action, it becomes essential to promote and nurture hope [17]. In this research, the average score on the Climate Change Hope Scale was determined to be high at 39.73 ± 5.52. A study conducted in Sweden found that constructive hope has a positive impact on environmentally friendly behavior [35]. Sangervo et al. found climate anxiety and climate hope to be strongly related to each other [36]. Experts have recently begun exploring ways to transform the helplessness created by climate change into hope and to enhance individuals’ awareness of climate change in order to support environmentally friendly behaviors [37]. In a study conducted with young people in Australia, participants mentioned feeling hopeless and powerless because they believed they were not in a position to mitigate the effects of climate change [38]. Therefore, it is necessary and desirable for nurses to be hopeful, both in alleviating their own climate concerns and in actively engaging in climate change prevention. The role of hope in relieving climate anxiety was discussed in the literature, but no study was found that emphasizes the role of the nurse in this. It is thought that the findings of this research contribute to the literature in this respect.

In the study, it was determined that there was a positive, statistically significant relationship between climate change worry and age, and between climate change worry and the hope scale score to prevent climate change. Li and Monroe found that climate anxiety has a direct effect on hope [16]. According to the study on nursing students, a statistically significant positive correlation was found between the Climate Change Worry Scale and the Climate Change Hope Scale, which is parallel to our findings [15]. The study’s findings indicate that nurses who exhibit higher levels of climate concern also tend to possess greater levels of hope. This implies that nurses, rather than succumbing to passivity and pessimism driven by anxiety, have the capacity to actively engage in change with a sense of hope. Hope can serve as a motivating factor in addressing climate anxiety. It is thought that nurses should make activating interventions to increase hope. Moreover, there is an observed increase in the awareness and responsiveness of older adults to shifting climate patterns [39]. In our study, the increase in climate anxiety with age confirms this finding. It is recommended that necessary interventions be made to address the climate anxiety of older nurses.

## Conclusion

In this study, nurses were found to have a high level of climate change worry and climate change hope. A positive linear relationship was observed between age and climate change worry. Similarly, a positive linear relationship was identified between climate change worry scale and climate change hope. Nurses are healthcare professionals who can provide mental support to individuals negatively affected by climate change. Additionally, they have significant responsibilities in educating individuals about potential harms of climate change, reducing environmental risks that can contribute to climate change, and actively participating in protecting individuals from the potential consequences of climate change. To be able to help others, nurses should first address their own climate concerns and maintain hope in climate change prevention. Therefore, it is considered important to provide support to nurses in this regard.

## Data Availability

The datasets used and/or analysed during the current study are available from the corresponding author on reasonable request.

## References

[1] Kahraman S, Şenol P. İklim Değişikliği: Küresel, Bölgesel ve Kentsel Etkileri. Acad Soc Sci J [Internet]. 2018;9789264091:353–70. https://dergipark.org.tr/en/pub/asj/issue/40224/479040.

[2] Brown MJ, White BP, Nicholas PK. Mental Health Impacts of Climate Change: Considerations for Nurse Practitioners. J Nurse Pract [Internet]. 2022;18(4):359–63. 10.1016/j.nurpra.2021.07.013.

[3] Turan E (2017). The Turkish Journal of Occupational/Environmental Medicine and Safety. TURJOEM.

[4] Aslan M, Yıldız A (2019). How blameless are hospitals in climate change? An example of a province in Turkey. Environ Socio-Economic Stud.

[5] Catton H (2020). Global challenges in health and health care for nurses and midwives everywhere. Int Nurs Rev.

[6] Leffers J, Butterfield P. Nurses play essential roles in reducing health problems due to climate change. Nurs Outlook [Internet]. 2018;66(2):210–3. 10.1016/j.outlook.2018.02.008. 10.1016/j.outlook.2018.02.008 29599047

[7] Benevolenza MA, DeRigne LA. The impact of climate change and natural disasters on vulnerable populations: A systematic review of literature. J Hum Behav Soc Environ [Internet]. 2019;29(2):266–81. 10.1080/10911359.2018.1527739.

[8] Ojala M, Cunsolo A, Ogunbode CA, Middleton J (2021). Anxiety, worry, and grief in a time of Environmental and Climate Crisis: a narrative review. Annu Rev Environ Resour.

[9] Bouman T, Verschoor M, Albers CJ, Böhm G, Fisher SD, Poortinga W et al. When worry about climate change leads to climate action: How values, worry and personal responsibility relate to various climate actions. Glob Environ Chang [Internet]. 2020;62(February):102061. 10.1016/j.gloenvcha.2020.102061.

[10] Kurt G, Akdur R (2024). Under what conditions does climate change worry contribute to Climate Action in Turkey. What Moderates this Relationship? Sustain.

[11] Stewart AE (2021). Psychometric properties of the climate change worry scale. Int J Environ Res Public Health.

[12] Innocenti M, Santarelli G, Faggi V, Ciabini L, Castellini G, Galassi F et al. Psychometric properties of the Italian version of the climate change worry scale. J Clim Chang Heal [Internet]. 2022;6:100140. 10.1016/j.joclim.2022.100140.

[13] Galway LP, Beery T, Buse C, Gislason MK (2021). What drives climate action in Canada’s provincial north? Exploring the role of connectedness to nature, climate worry, and talking with friends and family. Climate.

[14] Fertelli TK. Archives of Environmental & Occupational Health Awareness, worry, and hope regarding climate change among nurses : A cross-sectional study. Arch Environ Occup Health [Internet]. 2023;78(7–8):413–22. 10.1080/19338244.2023.2278521. 10.1080/19338244.2023.2278521 37933873

[15] Mat S, Çalışkan B, Baştarcan C (2024). Worry and hope levels of nursing students about climate change: a cross-sectional study. J Psychiatr Nurs.

[16] Li CJ, Monroe MC. Exploring the essential psychological factors in fostering hope concerning climate change. Environ Educ Res [Internet]. 2019;25(6):936–54. 10.1080/13504622.2017.1367916.

[17] Frumkin H, Hope. Health, and the Climate Crisis. J Clim Chang Heal [Internet]. 2022;5:100115. 10.1016/j.joclim.2022.100115.

[18] Luque-Alcaraz OM, Aparicio-Martinez P, Gomera A, Vaquero-Abellan M (2022). Nurses as agents for achieving environmentally sustainable health systems: a bibliometric analysis. J Nurs Manag.

[19] Kalogirou M, Olson J, Davidson S (2020). Nursing’s metaparadigm, climate change and planetary health. Nurs Inq.

[20] Liao W, Yang L, Zhong S, Hess JJ, Wang Q, Bao J et al. Preparing the next generation of health professionals to tackle climate change: Are China’s medical students ready? Environ Res [Internet]. 2019;168:270–7. 10.1016/j.envres.2018.10.006. 10.1016/j.envres.2018.10.006 30342323

[21] Portela Dos Santos O, Melly P, Joost S, Verloo H (2023). Climate Change, Environmental Health, and challenges for nursing Discipline. Int J Environ Res Public Health.

[22] Gaudreau C, Guillaumie L, Jobin É, Diallo TA. Nurses and climate change: a narrative review of nursing associations’ recommendations for integrating Climate Change Mitigation Strategies. Can J Nurs Res. 2024;08445621241229932. 10.1177/08445621241229932 PMC11308299 38373438

[23] Lopez-Medina IM, Álvarez-Nieto C, Grose J, Elsbernd A, Huss N, Huynen M et al. Competencies on environmental health and pedagogical approaches in the nursing curriculum: A systematic review of the literature. Nurse Educ Pract [Internet]. 2019;37(April):1–8. 10.1016/j.nepr.2019.04.004. 10.1016/j.nepr.2019.04.004 31002889

[24] Lira T, Ruth ML, Hannele T, Jouni J, Lauri K (2021). Finnish nurses’ perceptions of the health impacts of climate change and their preparation to address those impacts. Nurs Forum.

[25] Ergin E, Altinel B, Aktas E. A mixed method study on global warming, climate change and the role of public health nurses from the perspective of nursing students. Nurse Educ Today [Internet]. 2021;107(September):105144. 10.1016/j.nedt.2021.105144. 10.1016/j.nedt.2021.105144 34537496

[26] Kalogirou MR, Dahlke S, Davidson S, Yamamoto S (2020). Nurses’ perspectives on climate change, health and nursing practice. J Clin Nurs.

[27] Özbay S, Alci B (2021). Climate change worry scale: adaptation to Turkish, validity and reliability study. R&S. Resarch Stud Anatolia J.

[28] Gezer M, İlhan M (2020). İklim Değişikliğinin Önlenmesine Yönelik Umut Ölçeği: Türkçeye Uyarlama Çalışması. Mediterr J Educ Res.

[29] Clayton S, Karazsia BT. Development and validation of a measure of climate change anxiety. J Environ Psychol [Internet]. 2020;69(April):101434. 10.1016/j.jenvp.2020.101434.

[30] Tuckett A, Hegney D, Parker D, Eley RM, Dickie R (2011). The top eight issues Queensland Australia’s aged-care nurses and assistants-in-nursing worried about outside their workplace: a qualitative snapshot. Int J Nurs Pract.

[31] Wachholz S, Artz N, Chene D (2014). Warming to the idea: University students’ knowledge and attitudes about climate change. Int J Sustain High Educ.

[32] Hussey LK, Arku G. Are we ready for it? Health systems preparedness and capacity towards climate change-induced health risks: perspectives of health professionals in Ghana. Clim Dev [Internet]. 2020;12(2):170–82. 10.1080/17565529.2019.1610350.

[33] Demir R, Yalazı RÖ, Dinç A (2024). The relationship between women’s climate change awareness and concerns about climate change in Turkiye. Public Health Nurs.

[34] Kim Usher AM, Rice K, Fatema SR, Upward KL, Jones R. Nurses on the frontline of health care in the escalating context of climate change: climate-related extreme weather events, injustice, mental health and eco-anxiety. J Adv Nurs. 2023;(August). 10.1111/jan.15838 PMC12623706 37641422

[35] Ojala M (2012). Hope and climate change: the importance of hope for environmental engagement among young people. Environ Educ Res.

[36] Sangervo J, Jylhä KM, Pihkala P (2022). Climate anxiety: conceptual considerations, and connections with climate hope and action. Glob Environ Change.

[37] Çıplak E (2022). The mediating role of the future time perspective in the relationship between global climate change awareness and hope for the prevention of climate change. South Afr J Psychol.

[38] Gunasiri H, Wang Y, Watkins E, Capetola T, Henderson-wilson C, Hope (2022). Coping and Eco-anxiety: Young people’ s Mental Health in a climate-impacted Australia. Int J Environ Res Public Heal.

[39] Gifford E, Gifford R. The largely unacknowledged impact of climate change on mental health. Bull At Sci [Internet]. 2016;72(5):292–7. 10.1080/00963402.2016.1216505.

